# Optimization of antiplatelet/antithrombotic therapy for secondary stroke prevention

**DOI:** 10.4103/0972-2327.61270

**Published:** 2010

**Authors:** Padma Srivastava

**Affiliations:** Department of Neurology, CN Center, AIIMS, Ansari Nagar, New Delhi, India

**Keywords:** Antiplatelets, stroke, prevention

## Abstract

Role of antiplatelet therapy in secondary stroke prevention is of major significance. Antiplatelet agents predominantly in use are aspirin, clopidogrel, and combination regimes. The review focuses on the optimization of antiplatelet regimen based on evidence obtained from randomized-controlled trials, on different antiplatelet regimes and the risk assessment that may be unique to each patient.

Stroke is the second most common cause of death and disability today.[1] The World Health Organization estimates that 5.7 million people die from stroke each year. Among people 45- to 65 years old with an ischemic stroke, 8-12% will die within 30 days. The cumulative risk of a recurrent stroke in survivors is 7.7% at 1 year, and increases to 18.3% in 5 years.[1–2] Recurrent stroke risk after transient ischemic attack (TIA) or ischemic stroke ranges from 5% to 20% per year.[1–2] The highest risk is within the first few days after the initial event.[1–3] These staggering numbers emphasize the importance of managing the preventive aspect of stroke on an emergent basis. Numerous trials and meta-analysis have left no doubt that antiplatelet therapy effectively reduces stroke risk in patients with prior stroke or TIA. Nevertheless, several unanswered questions persist and despite the available recommendations, the preventive regimen in an individual patient remains largely “general.” Foremost among the controversies is the debate over optimization of antiplatelet regimen in an individual patient with unique set of risk factors.[4–5]

The majority of research in secondary stroke prevention supports the clinical value of aspirin. Aspirin has been most time-tested. Other regimes have been discovered, but aspirin remains the most non-controversial, inexpensive and reasonably effective (as compared with all other available regimes till date), albeit with a fraction lesser efficacy. The questions that remain unanswered satisfactorily are:

1. Should aspirin still be the most valued antiplatelet agent?
2. What is the optimal aspirin dosage?
3. Are combination antiplatelet agents with different modes of action better than a single agent?
4. Are the new antiplatlelet drugs like clopidogrel superior to aspirin?
5. Are more potent antiplatelet drugs e. g., GP IIbIIIa antagonists more effective/? safer than aspirin monotherapy?

## Antiplatelet Therapy

The Antiplatelet Trialists Collaboration (ATC)[6] published a meta-analysis of 287 trials with 135000 patients in comparison of antiplatelet therapy versus controls, and 77000 in comparison of different antiplatelet regimens. The main results were summarized as a reduction of serious vascular events (which include non-fatal MI, non-fatal stroke, or vascular death) by about 25%. In absolute terms the events prevented include 3.6% treated for 2 years. The absolute benefits substantially outweigh the absolute risks of major intracranial bleeding.

## Mechanism of Action of Antiplatelet Agents

To be considered as an antiplatelet agent, the following characteristics apply:

1. Inhibition of a measurable property of platelets such as adhesion, retention, or aggregation;
2. Inhibition of platelet-induced thrombus formation;
3. Prolongation of survival of radioactively labeled platelets in clinical or experimental situations in which platelet survival may be decreased.

Each of these antiplatelet agents have a unique mechanism of action.

Aspirin is a cyclooxygenase inhibitor, whereas the thienopyridines, ticlopidine, and clopidogrel inhibit the binding of adenosine diphosphate to its platelet receptor. Dipyridamole is considered to be a weak antiplatelet agent, but it is thought to enhance the antithrombotic activity of the vessel (e.g., via potentiation of nitric oxide, anti-oxidant properties, and inhibition of smooth muscle cell proliferation).

## Aspirin

Aspirin, the most commonly used antiplatelet agent, has been available for a long time. Aspirin remains an inexpensive, relatively safe, effective, easy-to-use, and widely accessible therapy in our armamentarium for stroke and cardiovascular disease prevention.[7–9] In the past 10 to 15 years, several newer antiplatelet agents have been approved for stroke prevention. The emergence of these additional agents provides new choices for physicians who are practicing stroke prevention.

Aspirin remains the most widely studied drug and at doses of 75 to 150 mg and is atleast as effective as a higher daily doses in reducing stroke risk. In comparison with placebo, the relative risk reduction for stroke, MI, or vascular death, is 13%. Table 1 lists the dosages of aspirin tried in various disorders.

**Table 1:** Aspirin dosages used in various vascular disorders

Clinical condition	Minimum effective daily dose (mg)
Men at high cardiovascular risk	75
Hypertension	75
Stable angina	75
Unstable angina	75
Acute MI	160
TIA and ischemic stroke	50
Severe carotid artery stenosis	75
Acute ischemic stroke	160

Higher doses have been tested in other trials and not found to confer any greater risk reduction. The optimal dosage of aspirin therefore continues to be elusive and at best the consensus seems to be 150–300 mg/day in most clinical situations.

### Safety

Aspirin has a well-known safety profile. Gastrotoxicity such as in-digestion, nausea, heartburn, vomiting, and gastro-intestinal bleeding are well-defined side effects.[7] One of the more serious complications related to aspirin administration is hemorrhagic stroke. The risk of hemorrhagic stroke for patients receiving aspirin for recurrent stroke prevention appears to be low. This may vary by race-ethnic group. Asians and African Americans who are generally at higher risk of hemorrhagic stroke, may possibly be at higher risk of hemorrhagic stroke when taking aspirin.[7–8] It has been speculated that evidence of microbleeds detected on gradient-echo magnetic resonance imaging (GRE MRI) brain studies might also place one at higher risk for intra-parenchymal brain bleeding with aspirin administration.[8–9] Careful control of blood pressure should be emphasized in an attempt to reduce hemorrhagic stroke risk in persons receiving aspirin therapy.

## Ticlopidine

### Efficacy

Ticlopidine became a popular agent for the prevention of recurrent stroke in the early and mid 1990s,[10–11] but very soon declined in usage on account of its side effects profile, the need for frequent laboratory monitoring of the complete blood count with differential and platelet count for neutropenia and thrombocytopenia during the first 3 months of therapy and the discovery that thrombotic thrombocytopenic purpura (TTP) can occur with this drug.[10–11]

Two large scale randomized trials, CATS[10] and TASS[11] (Canadian American Ticlopidine study and Ticlopidine, Aspirin Stroke Study) showed a reduction of 23.3% of vascular outcomes in patients with major stroke and 12% reduction after minor strokes at 3 years in those receiving Ticlopidine. *Post hoc* analysis of the TASS data suggested that non-whites had about 10% fewer adverse events and a greater risk reduction for key vascular outcome events.[11] These data prompted that development of African American Antiplatelet Stroke Prevention Study (AAASPS), sponsored by the NINDS. The data analysis did not support preferential use of ticlopidine. Added to that were the hugely important safety concerns. All these findings have led to almost abandonment of the use of ticlopidine in secondary stroke prophylaxis.

### Safety

The main adverse events associated with ticlopidine are diarrhea, other GI symptoms, rash, neutropenia, and thrombocytopenia. Hence, it is mandatory to obtain blood counts every 2 weeks in the first 3 months of therapy. TTP although rare can be fatal. It is estimated to occur one per 2000 to 4000 users.[10–11]

## Clopidogrel

Clopidogrel is a thienopyridine derivative chemically related to ticlopidine, which is superior to aspirin in stroke prevention.[12–13] The clopidogrel versus aspirin in patients at risk of ischemic events (CAPRIE) study[13] is the largest study to date that has tested clopidogrel in stroke patients. It was a randomized double-blind study that compared the effects of 75 mg of clopidogrel with 325 mg of aspirin once daily. In all, nearly 20000 patients with previous stroke MI or vascular death were included. After a mean follow up period of 1.9 years, there was a significant absolute risk reduction of 8.7% in favor of clopidogrel. Thus, clopidogrel is slightly more effective than aspirin in preventing a composite end-point of vascular events. In the *post hoc* analyses of CAPRIE trial, the benefit of clopidogrel was demonstrated to be amplified among high-risk subgroups, including patients with a history of previous MI, stroke, and those receiving lipid-lowering therapy, patients with DM, prior cardiac surgery, clopidogrel produced a relative risk reduction of 14.9% versus aspirin for the primary CAPRIE end point.[13]

Taken together the absolute difference between clopidogrel and aspirin is very small. Hence, a general use of clopidogrel in stroke patients is probably not justified. Clopidogrel is the agent of choice in patients with contraindications to or adverse effects on aspirin or in cases of aspirin failure. Also, clopidogrel seems to have an edge over aspirin in terms of effectively presenting stroke in subgroups of patients with coronary events; those with peripheral vascular disease, in women and in patients stented for carotid artery disease.

### Safety

The main side effects of clopidogrel alone are rash and diarrhea. Neutropenia has not been a concern. TTP may infrequently occur, a causal relationship seems uncertain.

## Clopidogrel Plus Aspirin

The MATCH (Management of Atherothrombosis with Clopidogrel in High-Risk patients with recent TIA or ischemic stroke) trial was a randomized double-blind placebo-controlled trial that compared aspirin (875 mg/day) versus placebo added on to patients receiving clopidogrel (75 mg) in 7599 high-risk patients with recent ischemic stroke or TIA and with atleast one additional risk factor.[14] The duration of treatment and follow up was for 18 months. The primary end point was either ischemic stroke, MI, vascular death, or acute hospitalization for ischemic event. 15.7% on combination therapy and 16.7% on clopidogrel alone had a recurrent event, which was non-significant. But, life-threatening bleeding was significantly higher with combination therapy (2.6% vs 1.3%).[14] These results are in contrast with those achieved in studies on patients where primary qualifying event was coronary ischemia.[15–16] The main difference is a higher rate of cerebral bleeding in patients with a prior stroke with the combination therapy. The clopidogrel and aspirin for Reduction of Emboli in Symptomatic Cartoid Stenosis (CARESS) trial[17] showed that with combination therapy, the number of microemboli from symptomatic carotid artery stenosis was reduced as compared to aspirin alone. The duration of this trial was short.

## Dipyridamole Plus Aspirin

The European Stroke Prevention Study (ESPS) 2 was a randomized double-blind placebo controlled trial that compared aspirin alone (50 mg) daily, extended release dipyridamole alone (200 mg twice a day) to combined aspirin and ER dipyridamole and placebo in patients who had suffered either a TIA or stroke.[18] End point over 2 years’ follow up was either ischemic stroke or vascular death. The 2-year relative risk reduction of stroke in the aspirin plus dipyridamole group (37%) was significantly higher than in either the aspirin group (18.1%) or the dipyridamole group (16.3%). So the combination treatment doubled the effect of aspirin alone. The results from ESPRIT (European/Australasian Stroke Prevention in Reversible Ischaemia Trial),[19] published in The Lancet confirm that extended-release dipyridamole plus acetylsalicylic acid (ASA) is superior to acetyl salicylic acid (ASA) as an antithrombotic prevention treatment for stroke patients. The study showed a statistically significant 20% relative risk reduction of primary outcome events (nonfatal stroke, death from all vascular causes, nonfatal myocardial infarction, or major bleeding complication) in patients treated with extended-release dipyridamole plus ASA compared with patients treated with ASA alone.[19] ESPRIT, an independent investigator initiated prospective, multicentre, randomized, open-label, blinded endpoint study, was conducted in 79 centers in 15 countries and randomized a total of 2739 patients with transient ischemic attack (TIA) or minor ischemic stroke of presumed arterial origin.[19] Patients were randomized to ASA (30-325 mg daily) or extended-release dipyridamole (200 mg twice daily) plus ASA (30-325 mg daily). The primary outcome event was the composite of death from all vascular causes, nonfatal stroke, nonfatal myocardial infarction, or major bleeding complication, whichever happened first.

## And Then Came ProFESS!

The largest ever recurrent stroke prevention trial, PRoFESS, results were presented at European Stroke Congress in Nice last year. PRoFESS is a double blind placebo-controlled clinical trial performed at 695 sites in 35 countries, with more than 20000 patients randomized to receive extended-release dipyridamole (200 mg) plus ASA (25 mg) given twice a day or clopidogrel (75 mg once a day and simultaneous randomization to telmisartan and plcebo.[20] Average observation time was 2.5 years. Among those with recurrent strokes, there were 25 fewer patients with recurrent ischemic strokes with ER-DP plus aspirin compared to clopidogrel but 38 more patients with hemorrhagic strokes with ER-DP plus aspirin compared to clopidogrel. Rates for main secondary outcome of stroke, MI, or vascular death were similar (13.1% vs. 13.1%).[20]

This is the largest secondary stroke prevention trial till date. The combination of aspirin and extended-release (ER) dipyridamole did not meet prespecified criteria for noninferiority versus clopidogrel, but rates of recurrent stroke, the primary outcome, were similar between the groups.

Therefore, the study did not show that aspirin plus extended-release dipyridamole or clopidogrel is superior to the other in the prevention of stroke. The findings provide additional safety and efficacy data physicians need in making individual treatment decisions for prevention of recurrent stroke or the combined end point of stroke, myocardial infarction, or death from vascular causes in their patients with stroke.

On the basis of these results, although aspirin remains the most commonly prescribed antiplatelet agent in patients with stroke and cautious guideline committees have not strongly favored one of the newer agents over the other, low-dose aspirin plus extended-release dipyridamole became the preferred agent for clinicians determined to select the best of all possible evidence-based antiplatelet regimens for secondary stroke prevention. Indirect comparison of these trials logically suggested that the combination would be superior to individual agents. However, the results of the MATCH,[14] CHARISMA,[15–16] and PRoFESS[20] trials show that the “compelling logic of transitive property, so reliable in mathematics has little authority in the often-illogical world of clinical trials.”[20] The trials not only failed to show superiority for the combination, but in one trial proved less safe ((MATCH)[14] and failed even to reach the noninferiority margin (PRoFESS).[20]

In an era of comparative effectiveness, when multiple agents are compared, randomized trials often cannot be understood in isolation, but must be interpreted in the context of sometimes complex networks of similar or relevant evidence. However, the reduction of such networks to clinical recommendations is not always straightforward. This concept gains much more significance when risk stratification and risk assessment in an individual patient is considered to impact the effectiveness of a given antiplatelet regimen.

Current guidelines in Europe and the United States recommend that for antiplatelet therapy after a stroke, aspirin, aspirin plus dipyridamole, and clopidogrel are options for prevention of stroke recurrence, but there is no recommendation for the use of one of these agents over the others.

## GP IIb/IIIa Antagonists

In the Blockade of the Glycoprotein IIb/IIIa Receptor to Avoid Vascular Occlusion (BRAVO) trial, patients with vascular disease were randomized to lotrafiban 30 mg or 50 mg twice a day or placebo in addition to aspirin at a dose ranging from 75 mg to 325 mg per day.[21] Follow-up was for 2 years. The primary end-point was the composite of all-cause mortality, MI, stroke, and recurrent ischemia. Of 9190 patients enrolled from 23 countires and 690 hospitals, 41% had stroke at the time of entry, and 59% had CAD. Death occurred n 2.3% of aspirin alone group versus 3% of lotrafiban group. And the cause of excess death was vacular related. The BRAVO trial[21] clearly showed that a combination of aspirin plus GPIIbIIIa antagonist is not superior to aspirin monotherapy but carries a higher bleeding risk.

## Cilostazole

Results of a randomized pilot trial, Cilostazol vs. Aspirin for Secondary Ischemic Stroke Prevention (CASISP), suggest that cilostazol is as effective as aspirin in preventing recurrent stroke, with significantly lower rates of bleeding.[22] Cilostazol has been shown to delay the onset of atherosclerosis, protect endothelium, and inhibit the proliferation of arterial smooth-muscle cells. The multicenter, double-blind CASISP trial enrolled 720 consecutive patients within 1 to 6 months of an ischemic stroke. The primary end point was any recurrence of stroke, including ischemic and hemorrhagic stroke, or subarachnoid hemorrhage. A primary end-point event occurred in 12 patients in the cilostazol group and 20 in the aspirin group. The estimated hazard ratio, calculated with Kaplan-Meier curves (risk of primary end point in the cilostazol group vs. the aspirin group) was 0.62 (95% CI, 0.30 – 1.26; *P* = 0.185).[22] At the end of the study, there was a 38.1% reduction in the relative risk of the primary end point in the cilostazol group versus the aspirin group. In addition, the difference in symptomatic hemorrhage is an important distinction between the treatments. In all, brain bleeding events occurred in seven patients treated with aspirin versus one on cilostazol, a significant difference (*P* = 0.034).[22]

## Stroke Prevention in Patients with AF

Anticoagulation therapy is superior to antiplatelet therapy in preventing stroke in AF.[23] Combining aspirin with anticoagulation therapy in AF will increase the bleeding risk. The only situation where combination therapy might be needed is in the setting of AF plus percutaneous coronary intervention and or stents and or acute coronary syndrome.

The consensus statements regarding AF and stroke prevention are:

- Atrial fibrillation (AF) affects 5% of people older than 65 years.
- Among patients with AF, the risk of stroke averages about 5% per year.
- The risk of stroke increases cumulatively with increasing age, previous transient ischemic attack or stroke, hypertension, diabetes, impaired left ventricular function, and a large left atrium.
- Management aims to identify and treat the underlying cause, control the ventricular rate, restore and maintain sinus rhythm, and minimize the risk of stroke.
- Warfarin reduces the risk of stroke by about two-thirds, and aspirin by about one-fifth.
- The risk of anticoagulant-associated hemorrhage increases with serious concomitant disease, and with poorly controlled hypertension and poorly controlled anticoagulation.
- All patients with chronic AF should be considered for oral anticoagulant therapy, and the decision based on the balance between the risks of thromboembolism and bleeding.
- The recommended international normalized ratio (INR) is 2.0-3.0.

Treating 1000 “average” AF patients (i.e., those with a 5% per year risk of stroke) with warfarin prevents about 30 strokes and causes at least two episodes of major hemorrhage each year. Treating 1000 AF patients with aspirin prevents about 15 strokes each year.

## Unique Clinical Situations and Decision Making

### Carotid artery stenosis

Recent studies suggest that the risk of very early recurrent stroke is particularly high in patients with carotid artery stenosis,[24] suggesting that more aggressive antiplatelet therapy may be indicated in this patient group. Increasing evidence suggests that asymptomatic microembolic signals (MES), which may be considered an optimal *in vivo* surrogate marker, detected by transcranial Doppler ultrasound (TCD), may be useful as markers of risk in patients with carotid stenosis. During a single hour 's recording from the ipsilateral middle cerebral artery, asymptomatic embolization has been reported in ≈40% of patients with symptomatic carotid stenosis.[25–29] MES are more common in patient groups known to be at higher risk of recurrent stroke, including those with more recent symptoms,[27,30] plaque ulceration[27,31,32] tighter stenosis,[27] and symptomatic versus asymptomatic status. The CARESS[17] trial demonstrated that, in actively embolizing patients with recently symptomatic carotid stenosis, combination therapy with clopidogrel and aspirin is more effective than aspirin alone in reducing asymptomatic embolization. The differences from MATCH[14] emphasize the heterogeneity of stroke and therefore the need for examining the effect of therapies on different stroke subtypes.

### Cervical artery dissection

In younger stroke patients, cervical artery dissection (CAD) is considered among the most important stroke etiologies.[33] The recurrence rate of stroke in CAD is <1% per year,[34–36] except for familial cases. It is still debated whether in CAD patients anticoagulation or antiplatelet agents are superior, balancing risk, and benefits of either approach.[37–39] Anticoagulation is widely advocated.[40] However, evidence from randomized trials on the efficacy of this therapy is missing.[41–42]

The pathophysiology, clinical observations, and the systematic metaanalysis provide several putative arguments in favor as well as against immediate anticoagulation in CAD patients. Until evidence-based data are available, the Table 2 may be clinically useful for individual treatment allocations as it summarizes putative arguments in favor versus against immediate anticoagulation of CAD.

**Table 2:** Clinical features against or in favor of immediate anticoagulation in cervical artery dissection

Against	Comment
Severe strokes, i.e. NIHSS score ≥15	In analogy to findings of increased rate of symptomatic hemorrhagic transformation in severe strokes.
No brain imaging available	CAD can present with bleedings.
Accompanying intracranial dissection	Bleeding risk seems ↑in intracranial dissection, e.g. vertebral artery dissection.
Local compression syndromes without stroke/TIA	Subadventitial dissection may have less risk for ischemic events.
Concomitant diseases with increased bleeding risk (extra/intracranial)	Translating atrial fibrillation studies to CAD.
Insufficient intracranial collaterals	Delayed ICA occlusion under heparin.
In favor	Comment
HITS despite (dual) antiplatelets	HITS more frequent in patients with recurrent ischemia.
Occlusion/ pseudo-occlusion	Embolization may occur during recanalization.
Multiple TIAs/strokes affecting multiple regions (same circulation)	Clinical course may suggest repetitive emboli
Free-floating thrombus	Rare finding

TIA: transient ischemic attack; NIHSS, National Institutes of Health Stroke Scale; ICA: internal carotid artery; HITS: high-intensity transient signals on transcranial Doppler study.

### Pregnancy and peri-partum period

For pregnant women with an ischemic stroke or TIA and high-risk thromboembolic conditions such as known coagulopathy or mechanical heart valves, the following options may be considered:[43]

- Adjusted-dose UFH throughout pregnancy such as a subcutaneous dose every 12 h with APTT monitoring
- Adjusted-dose LMWH with factor Xa monitoring throughout pregnancy
- UFH or LMWH until Week 13, followed by warfarin until the middle of the third trimester, when UFH or LMWH is then reinstituted until delivery.

Pregnant women with lower-risk conditions may be considered for treatment with UFH or LMWH in the first trimester, followed by low-dose aspirin for the remainder of the pregnancy. This has Class IIb, Level C evidence.

### Peri-operative period

It is common that patients who are scheduled for surgery are treated with antiplatelet agents due to their wide indications. The management of these antiplatelet agents in the perioperative period has a dual perspective: the risk of bleeding when the patient is operated under the effect of the antiplatelet agents against the risk of thrombosis if it has been withdrawn. The main challenges for the anaesthesiologist and the surgeon include patients with a coronary stent (mainly, new drugeluting coronary stents), those undergoing urgent surgery and those undergoing high bleeding risk surgery.

Current recommendations include the maintenance of aspirin if possible throughout the perioperative period, in order to limit the risks of cardiological, vascular or neurological postoperative events, although this makes it necessary to assume a small risk for hemorrhagic complications in some patients.[44] Nevertheless, there are many circumstances that are not clear yet and, in this situation, it is crucial that patients are treated with a multidisciplinary approach (anaesthesiologists, surgeons, cardiologists and hematologists). The approach remains individualistic and surgeons may prefer withdrawing antiplatelet agents for a period of 3 days to a week prior to surgery and restart over a period of few days to a week post surgery. Most decisions are individualistic and depend on case-selection.

### Antiplatelet therapy after hypertensive intra-cranial hemorrhage

There are no recommended guidelines as to whether and when to start secondary stroke prophylaxis after hypertensive intracranial hemorrhage. Although the risk factors remain identical and the risk of recurrence of strokes which may be ischemic exists in these patients, the risk of recurrence of hemorrhage versus infarct in these patients; risk of hemorrhage due to antiplatelet agents remain nebulous at best. Most strokologists verbally promulgate use of antiplatelet agents on an average 6 months after the intracranial hemorrhage.

## Treatment Expenses for Different Antiplatelet Regimes

Aspirin is cheap, widely available, easy to administer, and relatively safe and effective. Although aspirin plus extended release dipyridamole, clopidogrel, and ticlopidine may provide added recurrent stroke prevention benefit over aspirin alone, the former drugs are far more expensive than aspirin and will vary in cost by region. Cost-effectiveness analyses, with their inherent limitations suggest that in high-risk subjects, aspirin plus extended release dipyridamole over aspirin, and clopidogrel over aspirin alone are recommended. However, debate continues to rage about whether aspirin or one of the other antiplatelet agents should be used as initial therapy for recurrent stroke prevention.

## Antiplatelet Resistance and Failure

Concepts of antiplatelet resistance and failure are evolving. For aspirin, resistance may be defined as persistence of platelet activation and adhesion (as determined by platelet function studies) despite the administration of the drug, and failure as the occurrence of recurrent stroke or TIA despite administration of aspirin.[45] Failure of an antiplatelet agent may occur for any number of reasons. These may include patient non-compliance, inadequate dosing of the agent, resistance to the agent etc.[46] More recently, several cardiovascular disease studies have suggested resistance to aspirin based on measurement of a urinary marker of *in vivo* thromboxane generation, 11-dehydrothromboxane B2.

## Risk Assessment and Stratification of Therapy

If we now assume that any drug or drug combination more effectively inhibiting platelet function is not more effective than aspirin monotherapy, is there a subgroup of patients who could still benefit from clopidogrel or benefit in a particular way from the aspirin + ER-DP, or aspirin+ clopidogrel combination? A *post hoc* analysis was conducted using data from the ESPS-2 study. The rates of annual strokes and vascular events were determined for the aspirin + ER-DP group and the aspirin only group and were stratified by risk subgroup and univariate risk factors.[47] The maximal possible score is 10. Patients with a low risk of 0-2 showed no difference between aspirin and ER-DP. In patients with a higher risk score, the combination is clearly superior to aspirin monotherapy. This result shows that stratification of patients according to risk of recurrent stroke will lead to different treatment regimens.

## Points to Ponder

### Are the antiplatelet regimes based on patient based assessment or blindly chosen based on trial results?

Trial results are mere guidelines. They cannot be extrapolated indiscriminately to every patient encountered in clinical practice. These drugs are sometimes given indiscriminately to all patients who might remotely respond irrespective of the original guidelines. The indiscriminate use may dilute any potential therapeutic effects and exposes patients to possible adverse side effects.

### Should asprin resistance and failure be assessed routinely in all patients?

Although complex biochemical tests to document aspirin resistance or failure may not be justified in all patients, these two parameters which may impact the outcome of prophylaxis must be considerd and whenever deemed necessary must be carried out.

### Must every patient who has to be placed on antiplatelets first be screened for cerebral “microbleeds” on GRE MRI of brain?

#### Optimization of antiplatelets

Cerebral micorbleeds demonstrated by gradient echo technology on MRI scanning in patients taking aspirin may also play a predictive role in subsequent cerebral hemorrhage. To reduce drug-related cerebral hemorrhage, screening for cerebral microbleeds might be helpful before long-term antiplatelet therapy is started, and aspirin should be selected cautiously, particularly in patients with microbleeds at many sites.

### Brain attacks are different from Heart Attacks

Unlike MI, stroke is a variegated entity, and the pathogenesis and therapeutic potential in cardiogenic and lacunar strokes are entirely different from those of cerebeal vasculitis. These completely diverse cerebrovascular pathogeneses behave differently and should be treated separately. The varied etiopathogenesis causing strokes may explain the differential response to antiplatelet regimes in individual patient.

### Is industry driving the clinicians to prescribe indiscriminately? How serious is the influence of pharmaceutical companies on medical prescribing?

The question of how serious is the influence of pharmaceutical companies in our prescription practices is extremely poignant and of enormous ethical relevance. This needs intense soul searching. No doubt, industry and clinical practice can and be encouraged to live amicably and be symbiotic. But the ethics and science behind each prescription cannot be compromised. Since there is no wonder drug available till date.

From time to time industry has tried to influence clinicians in their favor one way or the other with a proliferation of inducements. This trend has certainly wormed its way into the clinical practices and has raised alarm in institutes concerned with ethical medical practices. Hopefully, this trend will soon disappear.

## On the Horizon

### Newer antiplatelet drugs

Emerging antiplatelet therapies presently being evaluated for secondary prevention of atherothromboembolism include other P_2_Y_12_ ADP receptor antagonists (prasugrel, cangrelor, AZD 6140), thromboxane receptor antagonists (e.g., S18886-terutroban), and thrombin receptor (PAR-1) antagonists (e.g., SCH530348).[48]

### The polypill

Wald and Law evaluated published meta-analysis to devise a single composite pill a polupill that would simultaneously reduce four major cardiovascular risk factors: raised LDL, high blood pressure, platelet aggregability, and raised blood homocyteine levels.[49] The pill would contain a statin, an antihypertensive agent, aspirin, and a folic acid and would be targeted toward everyone aged > 55 years and those with previous cardiovascular disease. Inspite of the flurry of hostile correspondence which ensued thereafter, a visit to any acute coronary or acute stroke unit today will demonstrate that this policy is largely in general use!

## Conclusions

It is of major importance to identify subgroups of patients with a very high risk of recurrence. In this subgroup of patients, clopidogrel as well as the combination therapies may be more superior to aspirin monotherapy.
